# Determinants of cigarette smoking among school adolescents in eastern Ethiopia: a cross-sectional study

**DOI:** 10.1186/1477-7517-9-39

**Published:** 2012-12-10

**Authors:** Ayalu A Reda, Asmamaw Moges, Berhanu Yazew, Sibhatu Biadgilign

**Affiliations:** 1 Department of Public Health, College of Health Sciences, Haramaya University, P.O. Box 235, Harar, Ethiopia; 2 Department of Environmental Health Science, College of Health Sciences, Haramaya University, Harar, Ethiopia; 3 Department of Epidemiology and Biostatistics, College of Medical and Health Sciences, Jimma University, Jimma, Ethiopia

**Keywords:** Tobacco, Cigarettes, Use, Behavior, School, Adolescent, Ethiopia

## Abstract

**Background:**

The World Health Organization (WHO) attributes more than 4 million deaths a year to tobacco, and it is expected that this figure will rise to 10 million deaths a year by 2020. Moreover, it is now a growing public health problem in the developing world.

**Objective:**

To assess the prevalence of cigarette use and its determinant factors among high school students in eastern Ethiopia.

**Methods:**

A cross-sectional study was conducted using structured self-administered questionnaires among 1,721 school adolescents in Harar town, eastern Ethiopia. Univariate and multivariate logistic regression analyses were performed to examine associations.

**Results:**

The analysis revealed that prevalence of ever cigarette smoking was 12.2% (95% CI 10.8% - 13.9%). Reasons mentioned for smoking cigarettes were for enjoyment (113, 52.8%), for trial (92, 42.9%), and for other reasons (9, 4.3%). The main predictors of cigarette smoking were sex (OR 4.32; 95% CI 2.59-7.22), age (OR 1.20; 95% CI 1.05-1.38) and having friends who smoke (OR 8.14; 95% CI 5.19-12.70). Living with people who smoke cigarettes was not significantly associated with smoking among adolescents (OR 1.25; 95% CI 0.81-1.92).

**Conclusion:**

This study concluded that high proportion of school adolescents in Harar town smoked cigarettes. Sex, age and peer influence were identified as important determinants of smoking. There is a need for early cost-effective interventions and education campaigns that target secondary school students.

## Introduction

Smoking is currently considered one of the greatest problems in public health worldwide, and it is one of the most preventable causes of death. Globally, the use and sale of substances such as alcohol and tobacco is causing substantial levels of health problems [1]. The World Health Organization (WHO) attributes more than 4 million deaths a year to tobacco and this figure is expected to increase to 10 million deaths a year by 2020. Moreover, it is now a growing public health challenge in the developing world [2]. According to WHO estimates, approximately 47% of men and 12% of women smoke cigarettes worldwide in 2010 [3]. Citing the death of 5 million individuals worldwide every year due to smoking-related diseases, the WHO states that smoking should be considered a pandemic [3]. In the United States, each year, approximately 440,000 persons die of diseases attributable to cigarette smoking leading to 5.6 million years of potential life lost, $82 billion in lost productivity, and $75 billion in direct medical costs [4].

Cigarette smoking has been described as a “gate way” substance towards illicit drug use among adolescents [5]. The onset of tobacco use occurs primarily in early adolescence, a developmental stage that is far removed by several decades from the death and disability that are associated with smoking in adulthood [6]. Therefore, the fact that many adult smokers initiated their smoking habit as adolescents makes adolescence smoking a significant public health problem [7]. It is also important as it is associated with respiratory health effects such as the incidence and exacerbation of asthma [8]. Studies showed that national smoking prevalence among men in sub-Sahara Africa varies from 20% to 60% and the annual cigarette consumption rates are on the rise for both men and women [7]. According to a report from Kenya, 7.2% of school going adolescents smoke cigarettes [7]. According to Ethiopian Demographic and Health Survey (EDHS) 2005 report, among men with the age range of 15–49 years in Ethiopia, 9% of them smoke cigarettes. Even though, there is no complete data on the prevalence of smoking among women, EDHS reported that less than 2% of women in Ethiopia smoke cigarettes [9]. Abuse of drugs is believed to be extremely rare in Ethiopia but use of locally growing psycho-stimulants such as tobacco, khat and cannabis is growing [10]. The health hazards, determinants of these substances and prevalence of their use have not been well studied [11]. Therefore, the aim of this study is to describe the prevalence of tobacco use and its determinants among school adolescents in eastern Ethiopia.

## Methods

### Settings and study design

A cross-sectional study was conducted among adolescents in secondary- and high schools located in Harar town. The study was conducted from April 12 to 26, 2010. Harari Regional State is one of the nine regions in Ethiopia and Harar town is its capital city. It is located in the eastern part of Ethiopia which is 510 km from Addis Ababa. The town has seven secondary schools from grades 9 to 10 (five governmental and two private) and two high schools of grades 11 to 12 (one governmental and one private). There were a total of 6,523 students enrolled in these schools at the time of the study.

### Sample and data collection

A total of 1890 students were included in the study. All schools were used for drawing a sample of respondents and proportional stratified sampling technique was employed to obtain study units in which a proportionate number of students were selected from each school and grades based on enrollment size at the time of the study. Within grades, all students in the selected classes were invited to participate regardless of their age. The Global Youth Tobacco Survey (GYTS) questionnaire was provided to students. The data collection was supervised and coordinated by the principal investigator and field supervisors, who were lecturers/instructors in Haramaya University. The questionnaires were checked by field supervisors at the end of each day during the survey, for omissions and for coding responses.

### Questionnaire

The GYTS is a questionnaire developed by the Global Tobacco Surveillance System Collaborating Group whose purpose and aims are explained elsewhere [10,12]. The GYTS uses standardized items that may be modified to fit local settings of the countries where it is used. It aims to collect information on the prevalence of smoking and other forms of tobacco use among adolescence. The English version of the GYTS questionnaire was translated into Amharic, the official language of the study area, by a panel of experts fluent in the language. The Amharic language questionnaire was used to collect the data after being pre-tested on 95 students in schools located outside the study area.

### Statistical analysis

Data were coded, entered, cleaned, and analyzed using SPSS Version 15 statistical software. Descriptive statistics were conducted using frequencies and proportions. Bivariate and multivariate analyses were carried out using logistic regression to examine the relationship between the outcome variable (ever smoking) and selected determinant factors. Adjusted and unadjusted odds ratios (OR) and their 95% confidence intervals (CI) were used as indicators of the strength of association. A p-value of 0.05 or less was used as cut-off level for statistical significance.

### Operational definition

We used the following operational definitions. Ever smoker was defined as a student who had ever tried smoking cigarettes in the past (once or twice puff). Current smoker was defined as a student who had smoked cigarettes on one or more days in the preceding month (30 days) of the survey.

### Ethical consideration

Ethical clearance was obtained from Haramaya University, College of Health Sciences. A letter of cooperation was written from Haramaya University to school authorities. During distribution of questionnaires, students were informed that the information collected would be kept anonymous and the objective of the study was explained to the study participants to obtain their consent. The students were also briefed about the confidentiality of their response and the importance of providing correct and accurate information, and that participation was voluntary.

## Results

### Socio-demographic characteristics

From among a total of 1890 students supplied with questionnaires, 1721 students responded, in the study, providing a response rate of 91.1%. Of these 856 (50.1%) were males and 851 (49.9%) females. The mean (SD) age of the participants was 16.4 (1.60) years (Table 1).

**Table 1:** Socio- demographic characteristics of school adolescents in Harar town, eastern Ethiopia

**Variables**	**Frequency**	**Percent**
Sex		
Female	851	49.9
Male	856	50.1
Age		
15-19	1085	63.5
20-25	622	36.45
Religion		
Orthodox	901	52.8
Protestant	36	2.1
Catholic	549	32.2
Muslim	191	11.2
Others	29	1.7
Ethnicity		
Amhara	721	42.1
Oromo	461	26.9
Adere	175	10.2
Gurage	134	7.8
Tigre	135	7.9
Somali	32	1.9
Others	54	3.2
Grade		
9th	839	48.9
10th	394	23.0
11th	253	14.7
12th	230	13.4
Living with		
Parents	1288	92.5
Friends	27	1.9
Relatives	43	3.1
Alone	34	2.4

### Smoking status of study subjects

A total of 214 (12.4%; 95% CI 10.8% - 13.9%) of the participants had at least one puff of a cigarette (4.4% among females, 20.6% among males). The mean (SD) age of starting cigarette smoking was 15.4 years (2.04). Sixty nine (32.4%) smokers have friends who smoke cigarettes. Seventy two (4.2%) were daily smokers and they were smoking an average of 5.9 cigarettes per day and spent an average of 13.25 ($1.02) Ethiopian birr per week. Most of the smokers (204, 95.4%) had purchased cigarettes from shops by themselves while 2.6% (6) purchased through friends. Reasons mentioned for smoking cigarettes were for enjoyment (113, 52.8%), for trial (92, 42.9%), and for other reasons (9, 4.3%). Smokers reported to smoke cigarettes in public recreation areas like bar and restaurants (60, 28.2%), at home (57, 26.7%) and at school compound (52, 24.4%) respectively. Ninety two (43.2%) of the students who currently smoke cigarette have desire to stop smoking.

### Predictors of ever smoking cigarettes

Sex of students was significantly associated with smoking where males had four times higher odds of smoking compared to females (OR 4.32; 95% CI 2.59-7.22). Higher age (OR 1.20; 95% CI 1.05-1.38) and having friends who smoke cigarettes (OR 8.14; 95% CI 5.19-12.70) were also found to be a significant predictor of smoking. Although the bivariate analysis showed significant difference in smoking cigarette by grade level, religion, and living with persons who smoke cigarettes, this difference disappeared when adjusting for other variables in the multivariate logistic regression model (Table 2).

**Table 2:** Predictors of ever smoking among in-school adolescents in Harar town, eastern Ethiopia

**Explanatory variable**	**Unadjusted OR (95% CI)**	**P-value**	**Adjusted OR (95% CI)**	**P-value**
Sex				
Male	5.51 (3.81-7.96)	<0.001	4.32 (2.59-7.22)	<0.001
Female	1.0		1.0	
Age	1.33 (1.22-1.46)	<0.001	1.20 (1.05-1.38)	<0.01
Grade				
9th	0.52 (0.35-0.78)	0.002	1.31 (0.67-2.56)	0.43
10th	0.71 (0.45-1.10)	0.13	0.97 (0.49-1.87)	0.92
11th	0.69 (0.42-1.14)	0.15	1.22 (0.63-2.36)	0.55
12th	1.0		1.0	
Marital status				
Single	0.42 (0.254-0.685)	0.04	0.52 (0.22-1.21)	0.128
Married	1.0		1.0	
Religion				
Orthodox Christian	1.19 (0.36-4.02)	0.77	4.21 (0.49-36.02)	0.19
Protestant	3.81 (0.95-15.31)	0.06	9.92 (0.93-106.21)	0.06
Catholic	1.26 (0.37-4.29)	0.71	3.06 (0.35-26.65)	0.31
Muslim	0.90 (0.25-3.28)	0.86	3.78 (0.41-34.62)	0.24
Others	1.0		1.0	
Having friend who smokes cigarette				
Yes	10.13 (7.16-14.34)	0.002	8.14 (5.19-12.70)	<0.01
No	1.0		1.0	
Living with peoples who smokes cigarette				
Yes	2.37(1.75-3.20)	0.03	1.25 (0.81-1.92)	0.313
No	1.0		1.0	

## Discussion

The present study revealed that 12.2% (95% CI 10.8% - 13.9%) of students smoked cigarettes (4.4% among females, 20.6% among males) and the current smoking practice is 4.2%. This seems to be similar to findings in Ethiopia and elsewhere. In Ethiopia, a study conducted in Addis Ababa for the Global Youth Tobacco Survey the prevalence reported that 10.1% of students had ever smoked cigarettes (boys, 15.2%; girls, 5.7%) where as 2.9% currently smoke cigarettes (boys 4.4%; girls, 1.0%). About 13.6% of never smokers were likely to initiate smoking in the next year [13]. In the same study, the overall prevalence of ‘ever users’ of tobacco products was 13.9%, 20.5% among boys and 2.9% among girls. It was estimated in a previous survey among high school students in northwest Ethiopia that the lifetime prevalence of smoking was 13.1% and the current prevalence rate of smoking was 8.1% [14]. A study among junior collegiate students in Nepal in 2007, reported that the prevalence of ‘current use’ of tobacco products was 10.2% [15]. A study of young people aged 15 to 24 years in Addis Ababa showed a current smoking prevalence of 11.8% in males and 1.1% in females [16]. In a study of rural populations in southern Ethiopia in 2005, the percentage of ever-smokers among respondents aged 15 years or more was 5.8% for ever smoking and 4.4% for current smoking [17].

In this study, one of the predictors of cigarette smoking was sex whereby more percentage of males smoked cigarettes than females. According to a study in Jakarta (Indonesia), Guangdong (China) and Nepal, male predominance was reported in the habit of smoking, while in Zambia [18], in the Indian cities of Delhi and Goa, and in the Czech Republic, no gender differences have been observed [19]. Smoking is found to be strongly associated with male sex in almost all populations in studies conducted in Africa [17,20-24]. This may be because females are more socially restricted than male counter parts and mostly young people imitate and exercise what they observe from their elders, parents and friends. Furthermore, familial relationships including care and family related activities may protect females from involving in tobacco use [25].

In this study, we detected a significant positive association between smoking and age (OR 1.20). Similarly, previous studies elsewhere have reported an association between age and cigarette smoking, where as age increases the odds of smoking also tend to increase [17,24,26-28]. This might be because among adolescents, an increase in age might lead to an increased trial of risky practices and experiment with substances such as tobacco. The subjective reason given for ever cigarette smoking in this study are also in agreement with this conjecture where 42.9% reported to smoke for “trial” and 52.6% for “enjoyment” among others.

We found that having friends who smoked cigarettes had an eight fold increase in the odds of smoking compared to non-smoking friends (OR 8.14). Several studies have previously reported the association between adolescent smoking and having a friend who smoke. This is consistent with studies conducted in parts of Ethiopia like Gondar [29], Addis Ababa and Butajira [30] as well as elsewhere [24]. For instance the GYTS study among adolescents in Addis Ababa revealed that among participants most or all of whose friends are smokers, there was a more than 30-fold increase in the odds of smoking compared to those who had no smoking friends, while those who had some smoking friends had a 9-fold increase in the odds of smoking [20]. The report that current smoking is associated with having friends that smoke could indicate a role for peer pressure or that risk taking students who would like to try smoking might be befriending smokers. For instance a study by Bricker and colleagues [31] reported that smoking among adolescents’ friends could influence both the initiation of smoking and its maintenance. This is affirmed by the report that most students obtained their first cigarette from a friend, indicating that initiation of smoking is linked to social relationships [32]. Furthermore diverse psychosocial factors have been associated with the use of cigarettes by adolescents which include having smoking parents [20], teachers [15] and peer pressure [27,33]. Also leisure activities, especially the ones that involve the company of friends [26,34] were associated with higher odds of smoking.

### Strengths and limitations of the study

Our survey is not representative of all adolescents or age groups, and as it is conducted among students it might not necessarily be generalizable to adolescents not enrolled in secondary- or high schools. Secondly, the survey applies only to adolescences who attended classes on the day of the survey and who completed the GYTS questionnaires. Hence delinquent students might be different from the population studied as they are expected to have higher levels of substance use. However, as the response rate was high (more than 90%) among the students present at the time of the survey, bias attributable to non-response among the population studied are expected to be limited. Furthermore, since smoking status was not validated by using biomarkers such as nicotine levels in saliva or exhaled carbon monoxide, it is difficult to estimate the actual extent of under or over reporting of smoking. Furthermore, there may be recall biases as students may not recall whether or not they smoked within the past 30 days prior to the day of the study. Despite the above limitations, the study has strengthens. We employed a standardized questionnaire that enabled us to compare to other studies conducted in similar settings. The prevalence estimates obtained are likely to closely represent the smoking prevalence among school going adolescents as we employed probability methods for selecting the sample.

## Conclusions

This study concluded that high proportion of school adolescents in Harar town ever smoked cigarettes. A modest proportion of these were current smokers. Sex, age and peer influence were identified as important determinants of smoking. Most students use cigarettes for enjoyment and those who have friend who smoke cigarettes were more at risk. This finding indicates that there is a need for early cost-effective interventions and education campaigns that target pre-secondary and secondary school students. Attention should not only be confined to secondary school but extend to their place of residence so that influences in the home environment and social surroundings that contribute to substance use are also tackled.

## Competing interests

All authors declare that they have no conflict of interest associated with the publication of this manuscript.

## Authors' contributions

AAR conceived and designed the study and collected data in the field, performed analysis, Interpretation of data, draft and critical review of the manuscript. AM assisted with the design, interpretation of data and the critical review of the manuscript. BYW participated in design and performed analysis, interpretation of data, and draft the manuscript and critically reviewed the manuscript and SB performed analysis, interpretation of data, and draft the manuscript and critically reviewed the manuscript. All authors approved and read the final manuscript. All authors participated in critical appraisal and revision of the manuscript.
